# TP53 and MDM2 Gene Polymorphisms, Gene-Gene Interaction, and Hepatocellular Carcinoma Risk: Evidence from an Updated Meta-Analysis

**DOI:** 10.1371/journal.pone.0082773

**Published:** 2013-12-23

**Authors:** Qiliu Peng, Xianjun Lao, Zhiping Chen, Hao Lai, Yan Deng, Jian Wang, Cuiju Mo, Jingzhe Sui, Junrong Wu, Limin Zhai, Shi Yang, Xue Qin, Shan Li

**Affiliations:** 1 Department of Clinical Laboratory, First Affiliated Hospital of Guangxi Medical University, Nanning, Guangxi, China; 2 Department of Occupational Health and Environmental Health, School of Public Health at Guangxi Medical University, Nanning, Guangxi, China; 3 Department of Gastrointestinal Surgery, Tumor Hospital of Guangxi Medical University, Nanning, Guangxi, China; Fudan University, China

## Abstract

**Background:**

The association between TP53 R72P and/or MDM2 SNP309 polymorphisms and hepatocellular carcinoma (HCC) risk has been widely reported, but results were inconsistent. To clarify the effects of these polymorphisms on HCC risk, an updated meta-analysis of all available studies was conducted.

**Methods:**

Eligible articles were identified by search of databases including PubMed, Cochrane Library, EMBASE and Chinese Biomedical Literature database (CBM) for the period up to July 2013. Data were extracted by two independent authors and pooled odds ratio (OR) with 95% confidence interval (CI) was calculated. Metaregression and subgroup analyses were performed to identify the source of heterogeneity.

**Results:**

Finally, a total of 10 studies including 2,243 cases and 3,615 controls were available for MDM2 SNP309 polymorphism and 14 studies containing 4,855 cases and 6,630 controls were included for TP53 R72P polymorphism. With respect to MDM2 SNP309 polymorphism, significantly increased HCC risk was found in the overall population. In subgroup analysis by ethnicity and hepatitis virus infection status, significantly increased HCC risk was found in Asians, Caucasians, Africans, and HCV positive patients. With respect to TP53 R72P polymorphism, no significant association with HCC risk was observed in the overall and subgroup analyses. In the MDM2 SNP309–TP53 R72P interaction analysis, we found that subjects with MDM2 309TT and TP53 Pro/Pro genotype, MDM2 309 TG and TP53 Arg/Pro genotype, and MDM2 309 GG and TP53 Pro/Pro genotype were associated with significantly increased risk of developing HCC as compared with the reference MDM2 309TT and TP53 Arg/Arg genotype.

**Conclusions:**

We concluded that MDM2 SNP309 polymorphism may play an important role in the carcinogenesis of HCC. In addition, our findings further suggest that the combination of MDM2 SNP 309 and TP53 Arg72Pro genotypes confers higher risk to develop HCC. Further large and well-designed studies are needed to confirm this association.

## Introduction

Liver cancer, which consists predominantly of hepatocellular carcinoma (HCC), was the sixth most common cancer worldwide and the third most common cause of cancer mortality in 2008 [1]. In high-risk China, liver cancer was the third most common cancer with 402,000 new cases and the second most common cause of death from cancer with 372,000 deaths in 2008 [2]. Besides, HCC is the fastest growing cause of cancer-related deaths in men of USA [3]. Thus, liver cancer is a serious fatal disease worldwide and has caused serious damage to human health. HCC accounts for about 90% of all primary liver cancers, and there are marked variations among geographic regions, racial, and ethnic groups, and between men and women [4]. Most HCC cases (about 80%) occur in either sub-Saharan Africa or Eastern Asia, and China alone accounts for more than 50% of the world’s cases [4]. As a complex and multi-factorial process, hepatocellular carcinogenesis is still not fully understood [4], [5]. Previous epidemiological studies have identified that major risk factors for the development of HCC are chronic infection with hepatitis B virus (HBV) or hepatitis C virus (HCV), liver cirrhosis, habitual alcohol abuse, and exposure to aflatoxin B1 [4], [5]. However, most individuals with these known environmental risk factors never develop HCC while many HCC cases develop among individuals without those known risk factors, suggesting that genetic factors also play an important role in hepatocellular carcinogenesis [5].

TP53 is a tumour suppressor that plays an important role in cell cycle regulation and the maintenance of genome integrity [6], [7], [8]. TP53 mediates the cellular response to DNA damage via effects on gene transcription, DNA synthesis and repair, genomic plasticity and apoptosis. Functional polymorphisms of the TP53 gene which influence the above activities of TP53 protein might be associated with human susceptibility to cancer. A common single nucleotide polymorphism in codon 72 of TP53 (rs1042522) causes the Arg to Pro amino acid substitution, and the 72Arg allele shows more efficient in inducing apoptosis [9] and lower ability in inducing cell cycle arrest and DNA repair [10], [11]. In addition, the human homolog of mouse double minute 2 (MDM2), acting as a major negative regulator of the TP53 tumor suppressor protein, directly binds to the latter to inhibit its activity as a transcription factor, and ubiquitinates it enhancing its proteolytic breakdown [12]. One polymorphism in the promoter region of MDM2, a T to G change at nucleotide 309 in the first intron (rs2279744), was associated with the enhanced MDM2 expression, and then attenuated function of the TP53 protein. Taken together, the two polymorphisms TP53 R72P and MDM2 SNP309 can accelerate carcinogenesis directly by affecting TP53 function and indirectly by down-regulation of TP53 via overexpression of MDM2, respectively. Hence, it is biologically reasonable to hypothesize a potential relationship between the TP53 R72P and MDM2 SNP309 polymorphisms and HCC risk.

Over the last two decades, a number of case–control studies have been conducted to investigate the associations between TP53 R72P and MDM2 SNP309 polymorphisms and HCC risk, but the results remain controversial and inconclusive. With respect to MDM2 SNP309 polymorphism, a meta-analysis by Ma et al. [13] found that the MDM2 SNP309 polymorphism was associated with an increased HCC risk in Asians and Caucasians, however, they failed to include all eligible studies in the meta-analysis [14], [15], [16], which make their conclusions questionable. With respect to TP53 R72P polymorphism, two meta-analyses [17], [18] investigating the same hypothesis, quite similar in methods and performed almost at the same time, yielded different conclusions. Furthermore, the two previous meta-analyses did not cover all eligible studies. The exact relationship between genetic polymorphisms of TP53 R72P and MDM2 SNP309 and HCC susceptibility has not been entirely established. To provide the most comprehensive assessment of the associations between the TP53 R72P and MDM2 SNP309 polymorphisms and HCC risk, we performed an updated meta-analysis of all available studies. The meta-analysis presented in this study aims to assess whether TP53 R72P and MDM2 SNP309 polymorphisms associated with HCC risk and to investigate the possible combined effect between the MDM2 SNP309 and the TP53 R72P polymorphisms on HCC risk.

## Methods

### Search Strategy

This study was performed according to the proposal of Meta-analysis of Observational Studies in Epidemiology group (MOOSE) [19]. We conducted a comprehensive literature search in PubMed, Cochrane Library, Embase, and Chinese Biomedical Literature database (CBM) databases up to July 01, 2013 using the following search strategy: (“liver cancer”, “hepatocellular carcinoma” or “HCC”) and (“TP53”, “P53”, “codon 72”, “Murine double minute 2”, or “MDM2”). There was no restriction on time period, sample size, population, language, or type of report. All eligible studies were retrieved and their references were checked for other relevant studies. The literature retrieval was performed in duplication by two independent reviewers (Xue Qin and Qiliu Peng). When multiple publications reported on the same or overlapping data, we chose the most recent or largest population.

### Selection Criteria

Studies included in the meta-analysis were required to meet the following criteria: (1) Case–control studies which evaluated the association between TP53 R72P and/or MDM2 SNP309 polymorphisms and HCC risk; (2) used an unrelated case–control design; (3) had an odds ratio (OR) with 95% confidence interval (CI) or other available data for estimating OR (95% CI); and (4) control population did not contain malignant tumor patients. Conference abstracts, case reports, editorials, review articles, and letters were excluded.

### Data Extraction

Two investigators (Xue Qin and Qiliu Peng) independently extracted data from the eligible studies. Data extracted from eligible studies included the first author’s name, publication date, country of origin, ethnicity, genotyping method, matching criteria, source of control, HCC confirmation, QC when genotyping, total numbers of cases and controls and genotype frequencies of cases and controls. The hepatitis virus infection status was additionally recorded for the stratified analysis. Two investigators checked the data extraction results and reached consensus on all of the data extracted.

### Quality Score Assessment

Methodological quality was independently assessed by two reviewers (Xue Qin and Qiliu Peng), according to a set of predefined criteria (Table 1) based on the scale of Thakkinstian et al. [20]. The revised criteria cover the credibility of controls, the representativeness of cases, assessment of HCC, genotyping examination, Hardy-Weinberg equilibrium (HWE) in the control population, and association assessment. A third reviewer (Shan Li) was invited to the discussion if disagreement still existed. Scores ranged from 0 (lowest) to 12 (highest). Articles with scores less than 8 were considered “low-quality” studies, whereas those with scores equal to or higher than 8 were considered “high-quality” studies.

**Table 1:** Scale for quality assessment.

Criteria	Score
Representativeness of cases	
Selected from any population cancer registry	2
Selected from any gastroenterology/surgery service	1
Selected without clearly defined sampling frame or with extensive inclusion/exclusion criteria	0
Credibility of controls	
Population- or neighbor- based	3
Blood donors or volunteers	2
Hospital-based (cancer-free patients)	1
Healthy volunteers, but without total description	0.5
Gastroenterology patients	0.25
Not described	0
Ascertainment of hepatocellular carcinoma	
Histological or pathological confirmation	2
Diagnosis of hepatocellular carcinoma by patient medical record	1
Not described	0
Genotyping examination	
Genotyping done under “blinded” condition	1
Unblinded or not mentioned	0
Hardy-Weinberg equilibrium	
Hardy-Weinberg equilibrium in controls	2
Hardy-Weinberg disequilibrium in controls	1
No checking for Hardy-Weinberg disequilibrium	0
Association assessment	
Assess association between genotypes and hepatocellular carcinoma with appropriate statistics and adjustment for confounders	2
Assess association between genotypes and hepatocellular carcinoma with appropriate statistics without adjustment for confounders	1
Inappropriate statistics used	0

### Statistical Analysis

Summary odds ratios (ORs) and corresponding 95% confidence intervals (CIs) were estimated for each polymorphism in different comparison models, including allelic contrast, additive genetic models, recessive genetic model, and dominant genetic model.

The *Q* test and *I^2^* statistics were used to assess the statistical heterogeneity among studies [21], [22]. If the result of the *Q* test was *P_Q_* <0.1 or *I^2^*≥50%, indicating the presence of heterogeneity, a random-effects model (the DerSimonian and Laird method) was used to estimate the summary ORs [23]; otherwise, when the result of the *Q* test was *P_Q_* ≥0.1 and *I^2^*<50%, indicating the absence of heterogeneity, the fixed-effects model (the Mantel–Haenszel method) was used [24]. To explore the sources of heterogeneity among studies, we performed logistic metaregression and subgroup analyses. The following study characteristics were included as covariates in the metaregression analysis: genotyping methods (PCR-RFLP versus not PCR-RFLP), ethnicity (Caucasian population versus Asian population), quality score (high quality studies versus low quality studies), source of controls (Hospital-based versus Population-based), QC when genotyping (Yes versus no), and HCC confirmation (pathologically or histologically confirmed versus other diagnosis criteria). Subgroup analyses were conducted by ethnicity and hepatitis virus infection status. Galbraith plots analysis was performed for further exploration of the heterogeneity.

Sensitivity analysis was performed by sequential omission of individual studies. For each polymorphism, publication bias was evaluated using Begg’s funnel plot and Egger’s regression asymmetry test [25]. The HWE of the control population in each eligible study was tested using a goodness-of-fit Chi-square test. All analyses were performed using Stata software, version 12.0 (Stata Corp., College Station, TX). All *p* values were two-sided. To ensure the reliability and the accuracy of the results, two authors entered the data into the statistical software programs independently with the same results.

## Results

### Study Characteristics

Based on our search criteria, 25 studies relevant to the role of TP53 R72P and/or MDM2 SNP309 on HCC susceptibility were identified. Six of these articles were excluded including 3 publications containing overlapping data [26]–[28], and 3 were meta-analyses [13], [17], [18]. Manual search of references cited in the published studies did not reveal any additional articles. As a result, a total of 19 relevant studies including 16 English articles [14], [15], [27], [29]–[41] and 3 Chinese papers (one was a dissertation of postgraduate student) [16], [42], [43] met the inclusion criteria for the meta-analysis (Figure S1). The main characteristics of the studies were presented in Table 2. Among them, 5 studies evaluated the MDM2 SNP309 polymorphism, 9 studies evaluated the TP53 R72P polymorphism, and 5 studies evaluated TP53 R72P and MDM2 SNP309 simultaneously. Therefore, a total of 10 studies including 2,243 cases and 3,615 controls were available for the meta-analysis of MDM2 SNP309 polymorphism and 14 studies containing 4,855 cases and 6,630 controls were included for TP53 R72P polymorphism. The sample size in these studies varied considerably, ranging from 183 to 3,727 individuals. Of all the eligible studies, 2 were conducted in Caucasians, 7 were in Asians, and 1 was in Africans for MDM2 SNP309 polymorphism; 9 were conducted in Asians, 4 in Caucasians, and 1 was in Africans for TP53 R72P polymorphism. Three studies were population–based and 16 were hospital–based studies. Fourteen articles of all eligible studies used quality control when genotyping and 4 studies in the present meta-analysis did not provide definite criteria for the HCC diagnosis. Several genotyping methods were used, including PCR-RFLP, TaqMan assay, and MALDI-TOF. The genotype distributions of the controls in 2 studies were not consistent with HWE for MDM2 SNP309 polymorphism [30], [35] and 2 were not consistent with HWE for TP53 R72P polymorphism [36], [38].

**Table 2:** Characteristics of eligible studies.

First author (Year)	Country	Ethnicity	Sample size (case/control)	Genotyping methods	Matching criteria	Source of control	HCC confirmation	QC when Genotyping	SNPs	HWE(*P* value)		Quality scores
										SNP309	Arg72Pro	
Akkiz 2004	Turkey	Caucasian	110/110	PCR-RFLP	Age, gender, smoking, drinking	PB	HC	Yes	MDM2 SNP309	0.239	–	9
Leu 2009	China	Asian	58/138	PCR-RFLP	Ethnicity	HB	NA	Yes	MDM2 SNP309	**0.048**	–	5
Dharel 2006	Japan	Asian	187/296	TaqMan assay	Drinking	HB	HC	Yes	MDM2 SNP309	0.724	–	7
Ezzikouri 2011	Morocco	African	96/222	PCR-RFLP	Age, gender	HB	NA	Yes	MDM2 SNP309, p53 Arg72Pro	0.508	0.685	4
Tomoda 2012	Japan	Asian	258/199	MALDI-TOF	Age, gender, drinking	HB	HC	No	MDM2 SNP309	0.640	–	8
Vuolo 2011	Italy	Caucasian	61/122	PCR-RFLP	Age, gender	PB	PC	No	MDM2 SNP309, p53 Arg72Pro	0.127	0.428	7
Wang 2012	China	Asian	310/794	PCR-RFLP	Age, gender	HB	HC	Yes	MDM2 SNP309	0.111	–	7
Yoon 2008	Korea	Asian	287/296	PCR-RFLP	Gender	HB	HC	No	MDM2 SNP309, p53 Arg72Pro	**0.063**	0.978	8
Yang 2013	China	Asian	350/326	TaqMan assay	Age	HB	PC	Yes	MDM2 SNP309, p53 Arg72Pro	0.738	0.636	8
Jiang 2008	China	Asian	1375/2352	PCR-RFLP	Age, gender	HB	PC	Yes	MDM2 SNP309, p53 Arg72Pro	0.134	0.145	8
Sumbul 2012	Turkey	Caucasian	119/119	PCR-RFLP	Age, gender, smoking, drinking	HB	HC	Yes	p53 Arg72Pro	–	**0.022**	9
Leveri 2004	Italy	Caucasian	86/254	PCR-RFLP	NA	HB	HC	No	p53 Arg72Pro	–	0.301	6
Yu 1999	China	Asian	80/328	PCR-RFLP	Smoking, drinking	HB	PC	Yes	p53 Arg72Pro	–	**0.019**	6
Anzola 2003	Spain	Caucasian	97/111	PCR-SSCP	Region	HB	NA	No	p53 Arg72Pro	–	0.375	5
Zhang 2012	China	Asian	985/992	TaqMan assay	Age, gender	HB	PC	Yes	p53 Arg72Pro	–	0.898	8
Peng 2004	China	Asian	192/192	PCR-RFLP	Age, gender, ethnicity	HB	PC	Yes	p53 Arg72Pro	–	0.481	7
Son 2013	Korea	Asian	157/201	PCR-RFLP	Age, gender, drinking	HB	NA	Yes	p53 Arg72Pro	–	0.086	7
Xu 2011	China	Asian	501/548	PCR-RFLP	Age, gender, region	PB	PC	Yes	p53 Arg72Pro	–	0.359	11
Zhu 2005	China	Asian	469/567	PCR-RFLP	Smoking, drinking	HB	HC	Yes	p53 Arg72Pro	–	0.321	8.25

SNP, Single nucleotide polymorphism; HC, Histologically confirmed; PC, Pathologically confirmed; NA, Not available; QC, Quality control; PB, Population–based; HB, Hospital–based; HWE, Hardy–Weinberg equilibrium in control population; PCR–RFLP, Polymerase chain reaction-restriction fragment length polymorphism; MALDI-TOF, Matrix-assisted laser desorption/ionization-time of flight mass spectrometry; PCR-SSCP, Polymerase chain reaction-single strand conformation polymorphism.

### Meta-analysis Results

Table 3 lists the main results of this meta-analysis.

**Table 3:** Summary of the meta-analysis results for MDM2 SNP309 and TP53 Arg72Pro polymorphisms and HCC risk.

Comparison	Population	No. of studies	Test of association			Mode	Test of heterogeneity		
			OR	95% CI	*P* Value		*χ^2^*	*P_Q_*	*I^2^*
MDM2 SNP309									
G vs. T	Overall	10	1.371	1.153–1.631	0.000	R	39.11	0.000	77.0
	Caucasian	2	1.987	1.488–2.653	0.000	F	0.01	0.907	0.0
	Asian	7	1.249	1.039–1.501	0.018	R	26.58	0.000	77.4
	African	1	1.569	1.090–2.258	0.015	–	–	–	–
	HBV positive	4	1.207	0.933–1.563	0.153	R	16.88	0.001	82.2
	HCV positive	3	1.481	1.245–1.761	0.000	F	2.67	0.263	25.1
	High quality studies	5	1.467	1.284–1.674	0.000	F	3.47	0.483	0.0
GG vs. TT	Overall	10	1.831	1.300–2.579	0.001	R	34.56	0.000	74.0
	Caucasian	2	3.604	1.991–6.524	0.000	F	0.00	0.974	0.0
	Asian	7	1.539	1.070–2.212	0.020	R	23.98	0.001	75.0
	African	1	2.604	1.079–6.280	0.033	–	–	–	–
	HBV positive	4	1.416	0.864–2.322	0.168	R	14.82	0.002	79.8
	HCV positive	3	2.198	1.542–3.134	0.000	F	1.65	0.437	0.0
	High quality studies	5	2.096	1.567–2.803	0.000	F	2.39	0.665	0.0
TG vs. TT	Overall	10	1.416	1.126–1.780	0.003	R	20.48	0.015	56.1
	Caucasian	2	2.433	1.509–3.922	0.000	F	0.26	0.613	0.0
	Asian	7	1.242	0.989–1.560	0.062	R	11.32	0.079	47.0
	African	1	1.590	0.958–2.641	0.073	–	–	–	–
	HBV positive	4	1.145	0.938–1.398	0.184	F	5.60	0.133	46.4
	HCV positive	3	1.759	1.280–2.418	0.001	F	1.90	0.387	0.0
	High quality studies	5	1.571	1.225–2.014	0.000	F	2.97	0.562	0.0
GG vs. TG+TT	Overall	10	1.398	1.148–1.703	0.001	R	19.51	0.021	53.9
	Caucasian	2	2.112	1.271–3.507	0.004	F	0.10	0.755	0.0
	Asian	7	1.298	1.053–1.601	0.015	R	1.20	0.977	0.0
	African	1	2.081	0.897–4.827	0.088	–	–	–	–
	HBV positive	4	1.244	0.932–1.661	0.139	R	9.51	0.023	68.5
	HCV positive	3	1.507	1.147–1.980	0.003	F	0.94	0.626	0.0
	High quality studies	5	1.564	1.281–1.908	0.000	F	1.70	0.791	0.0
GG+TG vs. TT	Overall	10	1.577	1.209–2.058	0.001	R	31.08	0.000	71.0
	Caucasian	2	2.734	1.743–4.289	0.000	F	0.14	0.704	0.0
	Asian	7	1.377	1.036–1.830	0.028	R	19.71	0.003	69.6
	African	1	1.719	1.058–2.794	0.029	–	–	–	–
	HBV positive	4	1.289	0.883–1.881	0.188	R	11.31	0.010	73.5
	HCV positive	3	1.926	1.426–2.601	0.000	F	2.09	0.352	4.2
	High quality studies	5	1.765	1.394–2.235	0.000	F	2.70	0.610	0.0
p53 Arg72Pro									
Pro vs. Arg	Overall	14	1.045	0.938–1.164	0.424	R	38.51	0.000	66.2
	Caucasian	4	1.105	0.897–1.362	0.347	F	4.43	0.218	32.3
	Asian	9	1.017	0.899–1.151	0.788	R	30.97	0.000	74.2
	African	1	1.330	0.911–1.941	0.140	–	–	–	–
	HBV positive	7	1.041	0.856–1.267	0.686	R	27.03	0.000	77.8
	HCV positive	3	0.957	0.721–1.270	0.759	F	0.32	0.851	0.0
	High quality studies	7	1.106	0.964–1.270	0.151	R	26.86	0.000	77.7
ProPro vs. ArgArg	Overall	14	1.132	0.887–1.446	0.319	R	43.70	0.000	70.2
	Caucasian	4	1.454	0.864–2.446	0.159	F	4.85	0.183	38.2
	Asian	9	1.044	0.805–1.356	0.744	R	33.12	0.000	75.8
	African	1	2.304	0.954–5.234	0.046	–	–	–	–
	HBV positive	7	1.159	0.751–1.789	0.506	R	31.95	0.000	81.2
	HCV positive	3	1.169	0.606**–**2.255	0.640	F	0.43	0.805	0.0
	High quality studies	7	1.285	0.934–1.768	0.123	R	33.80	0.000	82.2
ArgPro vs. ArgArg	Overall	14	1.023	0.938–1.114	0.611	F	17.77	0.166	26.9
	Caucasian	4	0.984	0.743–1.302	0.907	F	3.82	0.282	21.5
	Asian	9	1.017	0.889–1.162	0.810	R	13.83	0.086	42.2
	African	1	0.973	0.576–1.647	0.920	–	–	–	–
	HBV positive	7	0.957	0.845–1.083	0.483	F	10.38	0.110	42.2
	HCV positive	3	0.802	0.545–1.180	0.262	F	0.04	0.980	0.0
	High quality studies	7	1.061	0.964–1.167	0.228	F	4.64	0.591	0.0
ProPro vs. ArgPro+ArgArg	Overall	14	1.129	0.909–1.402	0.273	R	43.69	0.000	70.2
	Caucasian	4	1.561	0.946–2.574	0.081	F	5.36	0.148	44.0
	Asian	9	1.041	0.835–1.297	0.721	R	31.06	0.000	74.2
	African	1	2.327	0.949–5.162	0.038	–	–	–	–
	HBV positive	7	1.202	0.812–1.780	0.357	R	32.67	0.000	81.6
	HCV positive	3	1.291	0.684–2.437	0.430	F	0.43	0.807	0.0
	High quality studies	7	1.223	0.915–1.635	0.174	R	37.19	0.000	83.9
ProPro+ArgPro vs. ArgArg	Overall	14	1.032	0.912–1.167	0.619	R	23.41	0.037	44.5
	Caucasian	4	1.040	0.795–1.361	0.774	F	3.69	0.297	18.7
	Asian	9	1.020	0.878–1.184	0.799	R	19.36	0.013	58.7
	African	1	1.174.	0.725–1.900	0.515	–	–	–	–
	HBV positive	7	0.980	0.790–1.215	0.852	R	14.70	0.023	59.2
	HCV positive	3	0.855	0.594–1.233	0.402	F	0.14	0.932	0.0
	High quality studies	7	1.056	0.964–1.156	0.240	F	9.70	0.138	38.1

OR, odds ratio; CI, confidence intervals; R, random effects model; F, fixed effects model; PB, Population–based; HB, Hospital–based.

**Figure 1:**
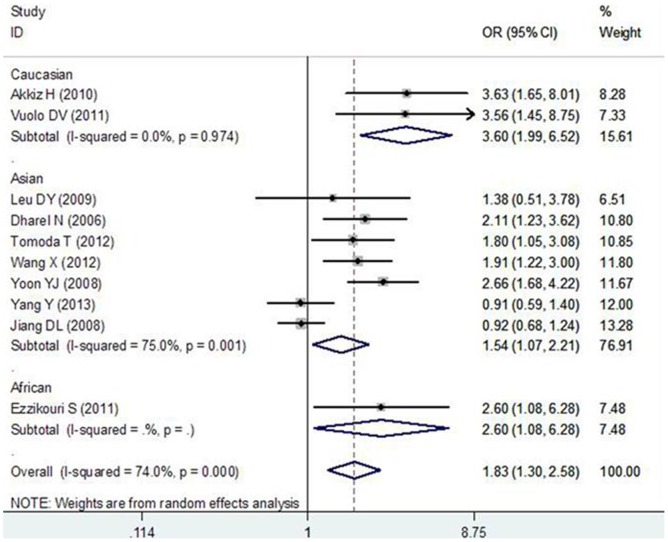
Subgroup analysis by ethnicity in the meta-analysis on the association between MDM2 SNP309 polymorphism and HCC risk using a random-effect model (additive model GG vs. TT).

For the MDM2 SNP309 polymorphism, the between-study heterogeneity was significant when all 10 studies were pooled into meta-analysis (I^2^>50.0%, or P_Q_ <0.10), thus, the random-effects model was used to pool the results. The results of pooling all studies showed that the MDM2 SNP309 polymorphism was associated with increased HCC risk in all genetic models (G vs. T: OR = 1.371, 95%CI: 1.153–1.631, P = 0.000; GG vs. TT: OR = 1.831, 95%CI: 1.300–2.579, P = 0.001, Figure 1; TG vs. TT: OR = 1.416, 95%CI: 1.126–1.780, P = 0.003; GG vs. TG+TT: OR = 1.398, 95%CI: 1.148–1.703, P = 0.001; GG+TG vs. TT: OR = 1.577, 95%CI: 1.209–2.058, P = 0.001). In subgroup analyses by ethnicity, the results showed that the MDM2 SNP309 polymorphism was associated with increased HCC risk in Asians (G vs. T: OR = 1.249, 95%CI: 1.039–1.501, P = 0.018; GG vs. TT: OR = 1.539, 95%CI: 1.070–2.212, P = 0.020; GG vs. TG+TT: OR = 1.298, 95%CI: 1.053–1.601, P = 0.015; GG+TG vs. TT: OR = 1.377, 95%CI: 1.036–1.830, P = 0.028), Caucasians (G vs. T: OR = 1.987, 95%CI: 1.488–2.653, P = 0.000; GG vs. TT: OR = 3.604, 95%CI: 1.991–6.524, P = 0.000; TG vs. TT: OR = 2.433, 95%CI: 1.509–3.922, P = 0.000; GG vs. TG+TT: OR = 2.112, 95%CI: 1.271–3.507, P = 0.004; GG+TG vs. TT: OR = 2.734, 95%CI: 1.743–4.289, P = 0.000), and Africans (G vs. T: OR = 1.569, 95%CI: 1.090–2.258, P = 0.015; GG vs. TT: OR = 2.604, 95%CI: 1.079–6.280, P = 0.033; GG+TG vs. TT: OR = 1.719, 95%CI: 1.058–2.794, P = 0.029). In subgroup analysis by hepatitis virus infection status, the results showed that the MDM2 SNP309 polymorphism was associated with increased HCC risk in HCV positive patients (G vs. T: OR = 1.481, 95%CI: 1.245–1.761, P = 0.000; GG vs. TT: OR = 2.198, 95%CI: 1.542–3.134, P = 0.000; TG vs. TT: OR = 1.759, 95%CI: 1.280–2.418, P = 0.001; GG vs. TG+TT: OR = 1.507, 95%CI: 1.147–1.980, P = 0.003; GG+TG vs. TT: OR = 1.926, 95%CI: 1.426–2.601, P = 0.000) but not in HBV positive subjects.

**Figure 2:**
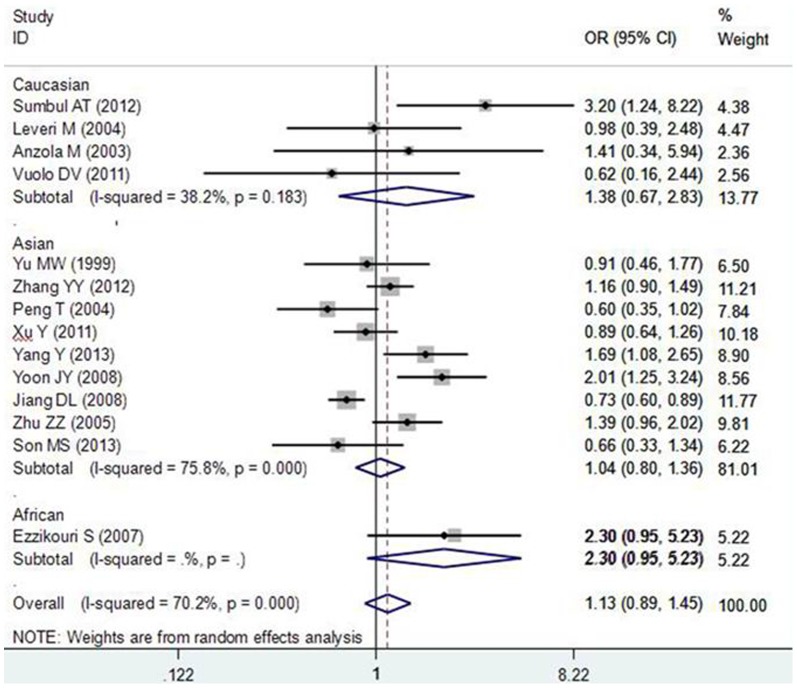
Subgroup analysis by ethnicity in the meta-analysis on the association between TP53 Arg72Pro polymorphism and HCC risk using a random-effect model (additive model ProPro vs. ArgArg).

For the TP53 R72P polymorphism, the between-study heterogeneity was also significant when all 14 eligible studies were pooled into meta-analysis (I^2^>50.0%, or P_Q_ <0.10), thus the random-effects model was used to pool the results. The meta-analysis results showed that the TP53 R72P polymorphism was not associated with increased HCC risk in the overall populations. In the stratified analyses by ethnicity and hepatitis virus infection status, statistically significant associations were also not observed in all subgroups (Figure 2).

For the MDM2 SNP309–TP53 R72P interaction analysis, the between-study heterogeneity was not significant in most of subgroups when all three eligible studies were pooled into meta-analysis (I^2^≤50.0%, and P_Q_ >0.10; Table 4), thus the fix-effects model was used to pool the results. In comparison to the reference MDM2 309TT and p53 Arg/Arg genotype, subjects with the MDM2 309TT and TP53 Pro/Pro genotype (OR = 1.996, 95%CI: 1.076–3.700, P = 0.028), MDM2 309 TG and TP53 Arg/Pro genotype (OR = 1.627, 95%CI: 1.110–2.385, P = 0.013), and MDM2 309 GG and TP53 Pro/Pro genotype (OR = 5.237, 95%CI: 2.845–9.639, P = 0.000) present significantly higher risk of developing HCC. However, no any dose-effect relationship was found between the MDM2 SNP309 and TP53 R72P polymorphisms on HCC risk.

**Table 4:** Summary odds ratios with confidence intervals for joint effect of MDM2 SNP309 and TP53 R72P polymorphisms on HCC risk.

TP53 Arg72Pro	MDM2 SNP309	No. of studies	Test of association			Mode	Test of heterogeneity		
			OR	95% CI	*P* Value		*χ^2^*	*P_Q_*	*I^2^*
Arg/Arg	TT	3	Reference		–	–	–	–	–
	TG	3	1.996	1.076–3.700	0.028	F	1.92	0.382	0.0
	GG	3	1.674	0.770–3.641	0.194	R	5.11	0.078	60.8
Arg/Pro	TT	3	1.066	0.693–1.639	0.771	F	1.63	0.442	0.0
	TG	3	1.627	1.110–2.385	0.013	F	0.89	0.639	0.0
	GG	3	1.315	0.827–2.090	0.247	F	0.85	0.655	0.0
Pro/Pro	TT	3	1.996	1.076–3.700	0.028	F	1.92	0.382	0.0
	TG	3	1.462	0.882–2.422	0.141	F	0.45	0.798	0.0
	GG	3	5.237	2.845–9.639	0.000	F	3.56	0.169	43.8

*P_Q_,* P value of Q-test for heterogeneity test; R, random-effects model, F, fixed-effects model.

### Heterogeneity Analysis

**Figure 3:**
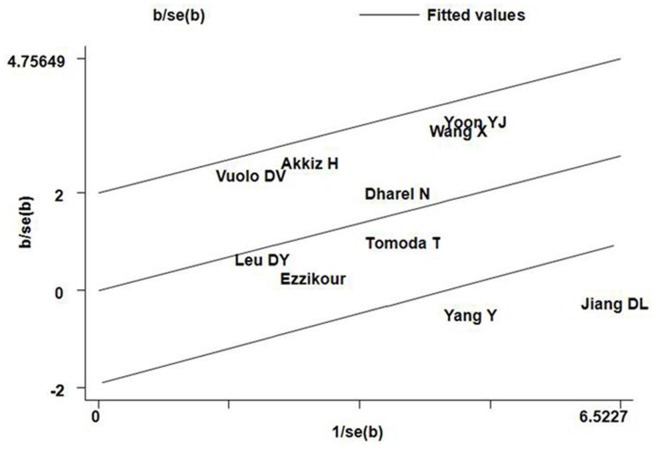
Galbraith plots of MDM2 SNP309 polymorphism and HCC risk (additive model GG vs. TT). The studies of Jiang et

For the MDM2 SNP309 polymorphism, the *I^2^* values of heterogeneity were greater than 50% and the *P_Q_* values were lower than 0.10 in all genetic comparison models (additive model GG vs. TT and TG vs. TT, recessive model GG vs. TG+TT, and dominant model GG+TG vs. TT) in the overall populations, which indicated statistically significant heterogeneity among studies. To explore the sources of heterogeneity, we performed metaregression and subgroup analyses. Metaregression analysis of data showed that the ethnicity and Quality scores were the major sources which contributed to heterogeneity (regression coefficient = 0.114, 95%CI: 0.075–0.153, p = 0.003 for ethnicity and regression coefficient = 0.217, 95%CI: 0.093–0.341, p = 0.016 for Quality scores, respectively). The Genotyping methods, HCC confirmation, Source of control, and QC when genotyping were not effect modifiers. Subsequently, we performed subgroup analyses by ethnicity and hepatitis virus infection status. However, heterogeneity still existed in all the above genetic comparison models in Asians and HBV positive subgroup (Table 3). To further investigate the heterogeneity, we performed Galbraith plots analysis to identify the outliers which might contribute to the heterogeneity. Our results showed that the studies Jiang et al. [16] and Yang et al. [15] were outliers in all the above four genetic comparison models in the overall populations (Figure 3). All *I^2^* values decreased obviously and *P_Q_* values were greater than 0.10 after excluding the two studies Jiang et al. [16] and Yang et al. [15] in all genetic comparison models in the overall populations, Asians and HBV positive subgroup. However, the significance of the summary ORs for the MDM2 SNP309 polymorphism in different comparison models in the overall populations, Asians and HBV positive subgroup were not influenced by omitting the two studies (Data not shown).

**Figure 4:**
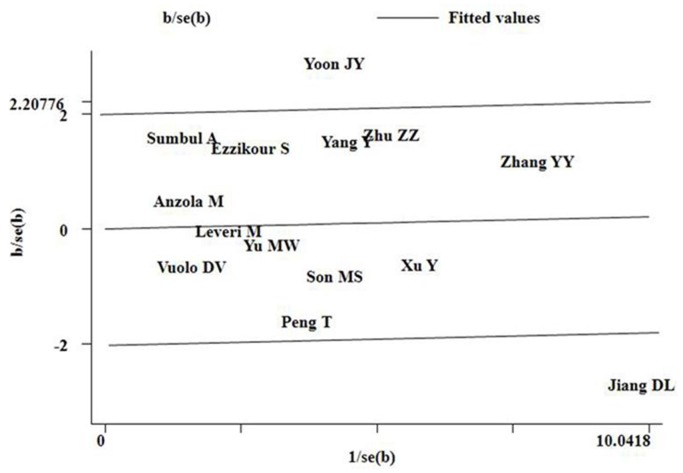
Galbraith plots of TP53 Arg72Pro polymorphism and HCC risk (additive model ProPro vs. ArgArg). The studies of Jiang et

For the TP53 R72P polymorphism, significant between-study heterogeneity was also observed in the pooling analyses of total available studies (the *I^2^* values of heterogeneity were greater than 50% and the *P_Q_* values were lower than 0.10 for additive model ProPro vs. ArgArg, recessive model ProPro vs. ArgPro+ArgArg, and dominant model ProPro+ArgPro vs. ArgArg). Metaregression analysis showed that the Quality scores was the major source heterogeneity (regression coefficient = 0.127, 95%CI: 0.051–0.203, p = 0.001). The Ethnicity, Genotyping methods, HCC confirmation, Source of control, and QC when genotyping were not effect modifiers. Galbraith plots analysis indicated that the studies by Jiang et al. [16] and Yoon et al. [35] were spotted as the major source of the heterogeneity (Figure 4). The *I^2^* values decreased obviously and *P_Q_* values were greater than 0.10 after excluding the two studies Jiang et al. [16] and Yoon et al. [35] in the overall populations, Asians and HBV positive subgroup. However, the significance of the ORs for the TP53 R72P polymorphism in the overall populations, Asians and HBV positive subgroup were also not changed by excluding the two studies.

### Sensitivity Analysis

Sensitivity analysis was performed by sequential omission of individual studies for both MDM2 SNP309 and TP53 R72P polymorphisms. For analyses of pooling more than three individual studies, the significance of ORs was not influenced excessively by omitting any single study (data not shown). For the MDM2 SNP309 polymorphism, sensitivity analysis was further performed by omitting the studies by Leu et al. [30] and Yoon et al. [35] in which the control populations were not in accordance with HWE. The significance of all ORs was not altered after excluding these two studies. For the TP53 R72P polymorphism, a sensitivity analysis was also further performed by omitting those two studies by Sumbul et al. [36] and Yu et al. [38] in which the control populations were deviated from HWE, and the significance of all ORs was also not altered.

### Publication Bias

Begg’s funnel plot and Egger’s test were performed to access the publication bias in this meta-analysis. Funnel plot shapes did not reveal obvious evidence of asymmetry, and all the p values of Egger’s tests were more than 0.05 for both MDM2 SNP309 and TP53 R72P polymorphisms, providing statistical evidence of the funnel plots’ symmetry (Figure S2). The results suggested that publication bias was not evident in this meta-analysis.

## Discussion

Previous studies investigating the associations between MDM2 SNP309 and/or TP53 R72P polymorphisms and HCC risk have provided inconsistent results, and most of those studies involved no more than a few hundred HCC cases, which is too few to assess any genetic effects reliably. Meta-analysis has been recognized as an important tool to more precisely define the effect of selected genetic polymorphisms on the risk for disease and to identify potentially important sources of between-study heterogeneity. Hence, we performed this meta-analysis including all available studies to provide the most comprehensive assessment of the associations between the MDM2 SNP309 and TP53 R72P polymorphisms and HCC risk. Our results suggested that the MDM2 SNP309 polymorphism was significantly associated with increased HCC risk in the overall populations, different ethnic subgroups, and HCV positive patients. However, our data did not support a genetic association between the TP53 R72P polymorphism and HCC risk. In the MDM2 SNP309–TP53 R72P interaction analysis, we found that individuals with MDM2 309TT and TP53 Pro/Pro genotype, MDM2 309 TG and TP53 Arg/Pro genotype, and MDM2 309 GG and TP53 Pro/Pro genotype had significantly increased HCC risk compared to those with the reference MDM2 309TT and TP53 Arg/Arg genotype.

Acting as a tumor suppressor, TP53 could lead to cell growth arrest and/or apoptosis in response to DNA damage and other cellular stresses [44]. The TP53 function is controlled by MDM2, which binds to TP53 and prevents TP53-dependent cell cycle arrest or apoptosis [45]. On the other hand, the MDM2 promoter is regulated by TP53 [46]. The functional TP53 Arg72Pro polymorphism has been shown to depress the activities of TP53 in inducing apoptosis, cell cycle arrest, and DNA repair [10], [11]. However, in the present meta-analysis, we found that TP53 R72P polymorphism was not significantly associated with the risk of HCC neither in the overall combined analysis nor the stratified analyses according to ethnicity and hepatitis virus infection status, which was inconsistent with the previous meta-analysis conducted by Lv et al. [17] and Ding et al. [18]. The main factor that would contribute to the discrepancy is that the previous meta-analyses with relatively small sample size may have insufficient statistical power to detect a real association with HCC risk or may have generated a fluctuated risk estimate. This observation is not a surprise because the TP53 72Pro allele could induce cell cycle arrest and DNA repair more efficiently to prevent transformation of normal cells [10].

MDM2 is one of the central nodes in the TP53 pathway. The proper regulation of MDM2 levels has been shown to be vital for TP53 tumor suppression, and even a modest change in levels could affect the TP53 pathway and, subsequently, cancer development in mouse models [47]. The study by Bond et al. [48] revealed that SNP309 GG cell lines expressed higher levels of MDM2 (on average 8-fold mRNA and 4-fold protein levels) than TT cell lines, whereas intermediate protein levels (on average 1.9-fold) were observed in heterozygous (TG) cell lines. Furthermore, Hong et al. [49] showed that SNP309 GG carriers had significantly higher MDM2 mRNA expression in esophageal tissue than TT carriers, but the TG heterozygote did not confer an increased MDM2 transcription. Thus, there is obvious biological evidence for the effects of MDM2 SNP309 polymorphism on HCC risk. In the present meta-analysis, we found that the MDM2 SNP309 polymorphism was significantly associated with increased HCC risk in the overall populations, different ethnic subgroups, and HCV positive patients. This functional relevance of MDM2 SNP309 polymorphism is consistent with the molecular epidemiological finding, demonstrating that the MDM2 SNP309 polymorphism played an important role in the HCC development.

HBV belongs to a family of DNA viruses called hepadnaviruses. The oncogenic potential of HBV has been attributed to its ability to integrate into host cellular DNA, which, may activate neighboring cellular genes directly to offer a selective growth advantage to the liver cells. In addition, production of hepatitis B x (HBx) protein can act as a transactivator on various cellular genes for cell growth and tumorigenesis [50]. In contrast, HCV is a positive-stranded RNA virus the genome of which does not seem to integrate into hepatocyte’s genome [51]. Therefore, differences in carcinogenetic mechanisms between these viruses may affect HCC development. In the present meta-analysis, we found that the MDM2 SNP309 polymorphism was significantly associated with increased HCC risk in HCV positive patients but not in HBV positive subgroup, which is consistent with the study conducted by Dharel et al. [31]. The explanation for preferentially increased HCC risk of MDM2 SNP309 polymorphism in HCV positive patients but not in HBV positive subgroup is that the TP53 function in the HCV patients could have been indirectly suppressed by the heightened MDM2 levels, making them more vulnerable to cancer development.

Previous research has demonstrated an interaction between MDM2 and TP53 at the molecular level [52], and the combined effects of MDM2 SNP 309 and TP53 Arg72Pro have been examined in lung cancer, Li–Fraumeni syndrome and non-polyposis colorectal cancer, with conflicting results [53], [54], [55]. In the present meta-analysis, we included all available three studies to explore the interaction effects between TP53 Arg72Pro and MDM2 SNP309 polymorphisms on HCC risk. We found that subjects with the MDM2 309TT and TP53 Pro/Pro genotype, MDM2 309 TG and TP53 Arg/Pro genotype, and MDM2 309 GG and TP53 Pro/Pro genotype present significantly increased risk of developing HCC as compared with the reference MDM2 309TT and TP53 Arg/Arg genotype. However, no any dose-effect relationship was found in the interaction analysis. To the best of our knowledge, this is the first meta-analysis to explore the combined effects of MDM2 SNP 309 and TP53 Arg72Pro polymorphisms on HCC risk. These results suggested a possible interaction effect between the MDM2 309GG and the TP53 72 Pro/Pro genotype in increasing the risk of HCC carcinogenesis.

Heterogeneity is a potential problem when interpreting the results of a meta-analysis, and finding the sources of heterogeneity is one of the most important goals of meta-analysis [56]. In the present meta-analysis, significant between-study heterogeneity in the pooled analyses of total eligible studies was observed (*P_Q_* values for MDM2 SNP 309 and TP53 Arg72Pro polymorphisms were all less than 0.10, and *I^2^* values were larger than 50.0%). To find the sources of heterogeneity, we performed subgroup analyses and. Subgroup analyses showed that the heterogeneity was still significant in Asians and HBV positive patients, while it was removed in the other subgroups, indicating that heterogeneity might result from the inconsistency of effects across those included studies from Asian population and HBV positive patients. Metaregression analysis showed that the ethnicity and Quality scores were the major sources of heterogeneity for MDM2 SNP 309 polymorphism and the Quality scores was the major source heterogeneity for TP53 Arg72Pro polymorphism. Subsequently, we performed Galbraith plots to further investigate the heterogeneity. For the MDM2 SNP 309 polymorphism, Galbraith plots spotted 2 studies [15], [16] as the outliers and the possible major sources of heterogeneity. For the TP53 Arg72Pro polymorphism, Galbraith plots also spotted 2 studies [16], [35] as the outliers and the possible major source of heterogeneity. Interestingly, the studies spotted as the outliers were all from Asian populations and HBV positive subgroup, which further confirmed that the inconsistency of effects across those studies from the above population might be the major sources of heterogeneity in this meta-analysis. When excluding the studies of Yang et al. [15] and Jiang et al. [16] for MDM2 SNP 309 polymorphism and the studies of Jiang et al. [16] and Yoon et al. [35] for p53 Arg72Pro polymorphism, all *I^2^* values decreased lower than 50% and *P_Q_* values were larger than 0.10 in all genetic comparison models in the overall populations, Asians, and HBV positive subgroup. However, the summary ORs for the MDM2 SNP309 and TP53 Arg72Pro polymorphisms in different comparison models in the overall population and subgroup analyses were not material change by omitting the studies spotted as outliers, indicating that our results were robust and reliable.

Some possible limitations in this meta-analysis should be acknowledged. First, the overall outcomes were based on individual unadjusted ORs, whereas a more precise evaluation should be adjusted by potentially suspected factors including age, gender, smoking status, and environmental factors. Second, the controls were not uniformly defined. Although most of the controls were selected mainly from healthy populations, some had benign disease such as liver cirrhosis, HBsAg positive and so on. Therefore, non-differential misclassification bias was possible because these studies may have included the control populations who have different risks of developing HCC. Third, the number of studies included in the meta-analysis for African population was relatively small and there was only one study in the African group, which may lead to low statistical power and generated fluctuate estimation. Finally, gene–environment interactions were not addressed in this meta-analysis due to the lack of sufficient data. As is generally accepted, aside from genetic factors and chronic infection with HBV or HCV, exposure to aflatoxin B1, liver cirrhosis, and habitual alcohol abuse are major risk factors for HCC; however, we could not perform subgroup analyses based on these environmental exposures owing to the limited reported information on such associations in those included studies.

Despite these limitations, this meta-analysis suggests that the MDM2 SNP 309 polymorphism but not TP53 Arg72Pro variant is associated with increased risk of HCC. In addition, our findings further suggest that the combination of MDM2 SNP 309 and TP53 Arg72Pro genotypes confers higher risk to develop HCC. However, it is necessary to conduct large sample studies using standardized unbiased genotyping methods, homogeneous HCC patients and well-matched controls. Moreover, gene–environment interactions should also be considered in the analysis. Such studies taking these factors into account may eventually lead to better, comprehensive understanding of the association between these polymorphisms and HCC risk.

## Supporting Information

Figure S1 Flow diagram of included studies for this meta-analysis.
(TIF)

Figure S2 Funnel plot analysis to detect publication bias. Each point represents a separate study for the indicated association. A Funnel plot for MDM2 SNP309 polymorphism in the overall analysis (recessive model GG vs. TG+TT: P = 0.180); B Funnel plot for TP53 Arg72Pro polymorphism in the overall analysis (recessive model ProPro vs. ArgPro+ArgArg: P = 0.114).
(TIF)

Checklist S1 Checklist of items to include when reporting a systematic review or meta-analysis.
(DOC)
