# Supplementary figures and images for: TP53 and MDM2 Gene Polymorphisms, Gene-Gene Interaction, and Hepatocellular Carcinoma Risk: Evidence from an Updated Meta-Analysis
Source: PLoS One. 2013 Dec 23;8(12):e82773. doi: 10.1371/journal.pone.0082773 (PMC3871586; doi:10.1371/journal.pone.0082773)

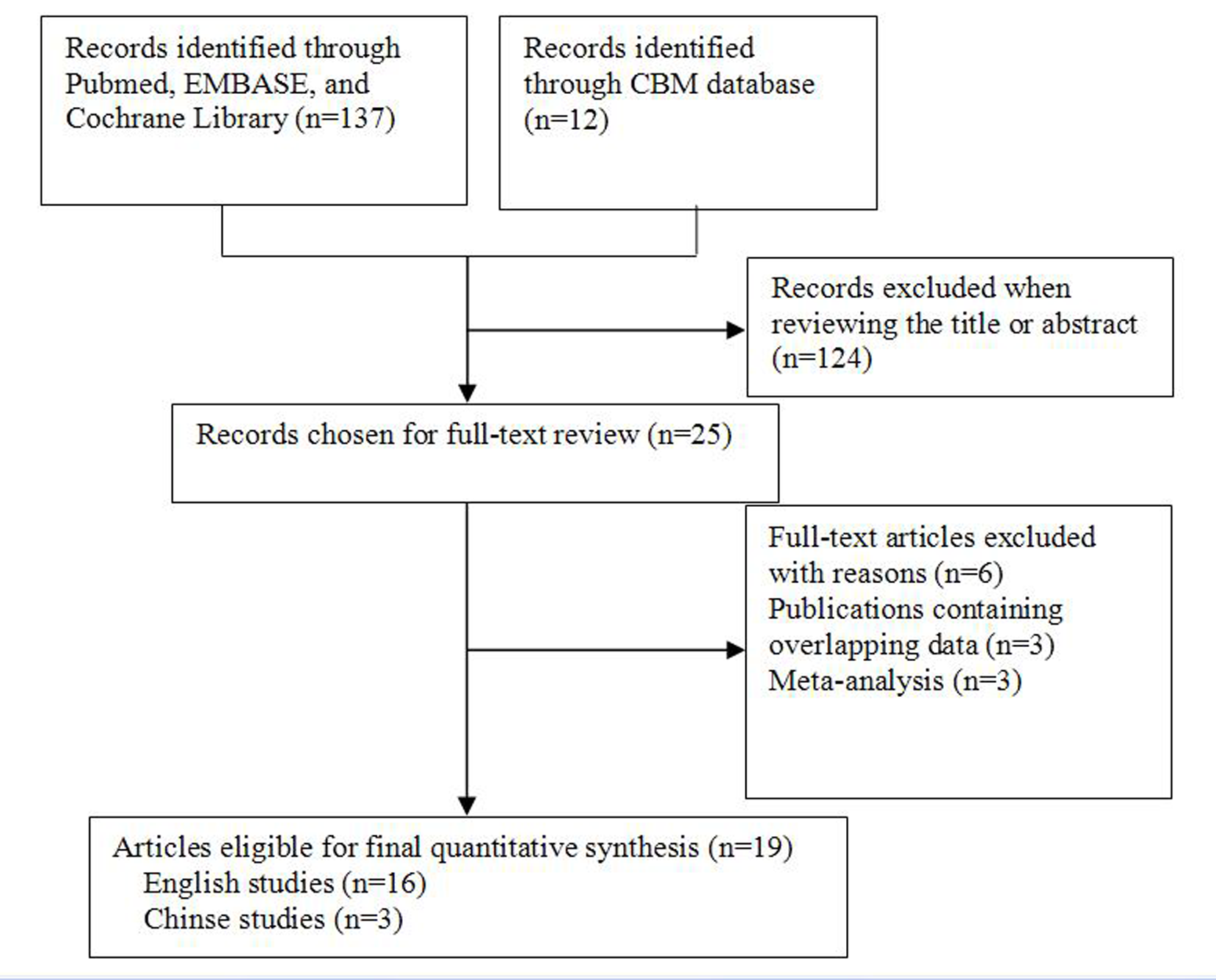

Supplement: Figure S1 — Flow diagram of included studies for this meta-analysis. (TIF) [file pone.0082773.s001.tif]

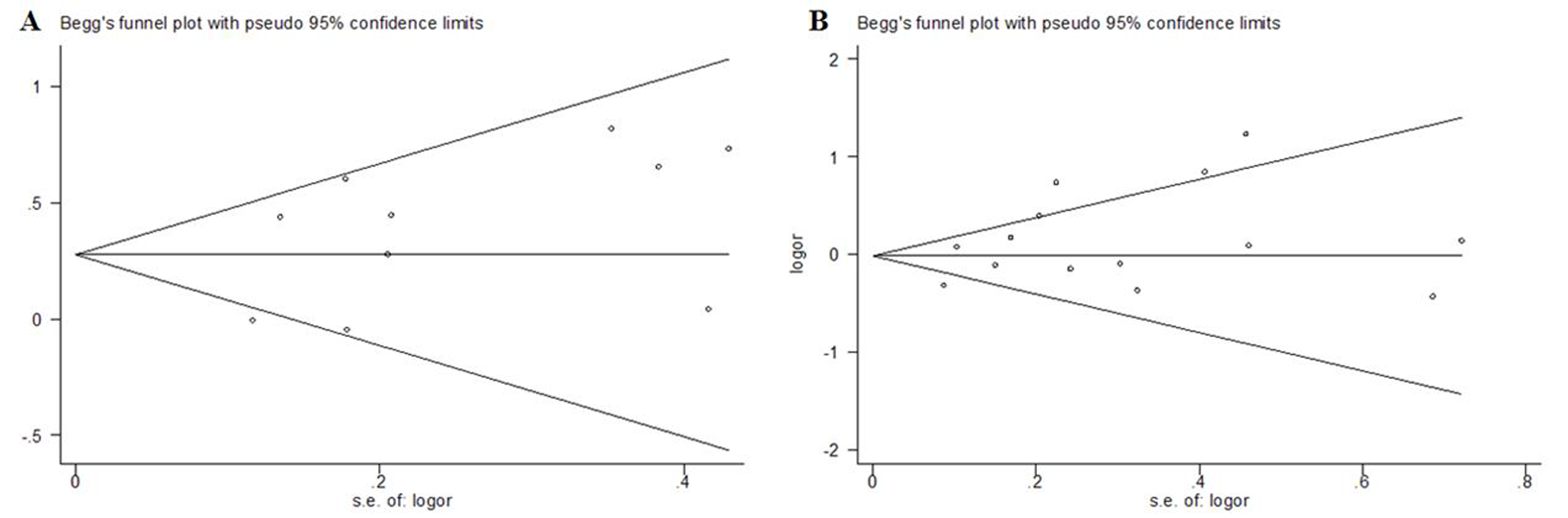

Supplement: Figure S2 — Funnel plot analysis to detect publication bias. Each point represents a separate study for the indicated association. A Funnel plot for MDM2 SNP309 polymorphism in the overall analysis (recessive model GG vs. TG+TT: P = 0.180); B Funnel plot for TP53 Arg72Pro polymorphism in the overall analysis (recessive model ProPro vs. ArgPro+ArgArg: P = 0.114). (TIF) [file pone.0082773.s002.tif]
